# Sex-specific associations of empirically derived dietary patterns with colorectal cancer risk in a Korean population: a case‒control study

**DOI:** 10.1038/s41598-024-55524-5

**Published:** 2024-03-20

**Authors:** Minji Kim, Madhawa Gunathilake, Jeonghee Lee, Jae Hwan Oh, Hee Jin Chang, Dae Kyung Sohn, Aesun Shin, Jeongseon Kim

**Affiliations:** 1 Department of Cancer Biomedical Science, National Cancer Center Graduate School of Cancer Science and Policy, Goyang-si, Gyeonggi-do South Korea; 2 Center for Colorectal Cancer, National Cancer Center Hospital, National Cancer Center, Goyang-si, Gyeonggi-do South Korea; 3 Department of Preventive Medicine, Seoul National University College of Medicine, Jongno-gu, Seoul, South Korea

**Keywords:** Cancer, Diseases, Health care, Oncology, Pathogenesis, Risk factors

## Abstract

Dietary patterns may be a crucial modifiable factor in colorectal cancer (CRC) risk. This study aimed to examine the associations of dietary patterns derived from two methods with CRC risk in Korea. In a study of 1420 CRC patients and 2840 control participants, we obtained dietary patterns by principal component analysis (PCA) and reduced rank regression (RRR) using 33 predefined food groups. The associations between dietary patterns and CRC risk were assessed using unconditional logistic regression models to calculate odds ratios (ORs) and 95% confidence intervals (CIs). We identified two similar dietary patterns, derived from PCA 1 (prudent) and RRR (healthy), characterized by higher consumption of green/yellow vegetables, light-colored vegetables, fruits, eggs, and milk in both men and women. In women, higher prudent and healthy pattern scores were significantly associated with a lower risk of CRC (prudent, OR_Q4 vs. Q1_ = 0.59, 95% CI 0.40–0.86, *P* for trend = 0.005; healthy, OR_Q4 vs. Q1_ = 0.62, 95% CI 0.43–0.89, *P* for trend = 0.007). In men, a significant inverse association between dietary pattern and risk of rectal cancer was found only for the healthy dietary pattern (OR_Q4 vs. Q1_ = 0.66, 95% CI 0.45–0.97, *P* for trend = 0.036). Compared with the dietary pattern derived by PCA, the RRR dietary pattern had a slightly stronger association with a lower risk of distal colon cancer (OR_Q4 vs. Q1_ = 0.58, 95% CI 0.35–0.97, *P* for trend = 0.025) and rectal cancer (OR_Q4 vs. Q1_ = 0.29, 95% CI 0.15–0.57, *P* for trend < 0.001) in women. Our findings suggest cancer prevention strategies focusing on a diet rich in vegetables, fruits, eggs, and milk. Moreover, the use of both PCA and RRR methods may be advantageous to explore the associations between dietary patterns and risk of CRC.

## Introduction

In 2020, the Global Cancer Observatory (GLOBOCAN) estimated that colorectal cancer (CRC) is one of the most commonly diagnosed cancers worldwide^1^. In Korea, cancer is the leading cause of death, accounting for 27.5% of all deaths in 2019. In particular, the incidence and mortality rates of CRC rank fourth and third, respectively, among all cancer types^2^. Based on previous epidemiological studies, diet and lifestyle factors have been recognized as key strategies for the primary prevention of CRC^3–5^. In recent nutritional epidemiological studies related to cancer, diet has been evaluated in a more complex manner rather than considering each diet or nutrient individually, which helps to understand dietary pattern concepts^6,7^.

The dietary pattern can be defined as combinations of dietary components intended to encompass the total diet in a specific population^8^. This approach is advantageous for exploring the combined effects and interactions of nutrients and food products. Although the components of dietary patterns differed across populations, the most widely investigated patterns in relation to cancer risk, especially CRC were prudent/healthy and western/unhealthy patterns^9^. Typically, a prudent dietary pattern is characterized by higher intakes of fruits and vegetables, and this pattern has been reported to lower the risk of developing CRC^10,11^. In contrast, a Western dietary pattern containing higher amounts of meat and processed foods has been shown to increase the risk of CRC^12,13^.

Several strategies have been applied to identify dietary patterns using hypothesis-driven methods or data-driven methods in relation to cancer, such as factor analysis, principal component analysis (PCA), reduced rank regression (RRR), partial least squares (PLS), and Gaussian graphical models (GGMs)^14–17^. Specifically, the use of PCA for identifying dietary patterns aims to explain the maximum variation in dietary intake and hence reflects the actual dietary behaviors within a population^18^. However, a major criticism of PCA is that this method does not necessarily identify a dietary pattern that is associated with the disease of interest. To determine disease-related dietary patterns, the RRR was proposed as an alternative to PCA^19^. The RRR aims to explain the maximum variation in investigator-specific intermediate response variables that are potentially relevant for a disease^20^, whereas dietary patterns derived using this method could be behaviorally irrelevant^21^. However, few studies have assessed the relationship between dietary patterns and CRC using the RRR method. A case‒control study revealed that an RRR-derived dietary pattern characterized by high intakes of grains, vegetables, and fruits was inversely associated with colon cancer^22^. However, another prospective cohort study reported that the empirical dietary inflammatory pattern score derived from the RRR was associated with an increased risk of developing CRC^23^. These contradictory results might be attributed to the selection of response variables that act as intermediates in the relationship between dietary intake and the disease of interest^24^.

Thus, we aimed to identify the major dietary patterns in a Korean population to determine the associations between those patterns and the risk of CRC using PCA and RRR methods. Moreover, we compared dietary patterns according to sex.

## Materials and methods

### Study population

**Figure 1:**
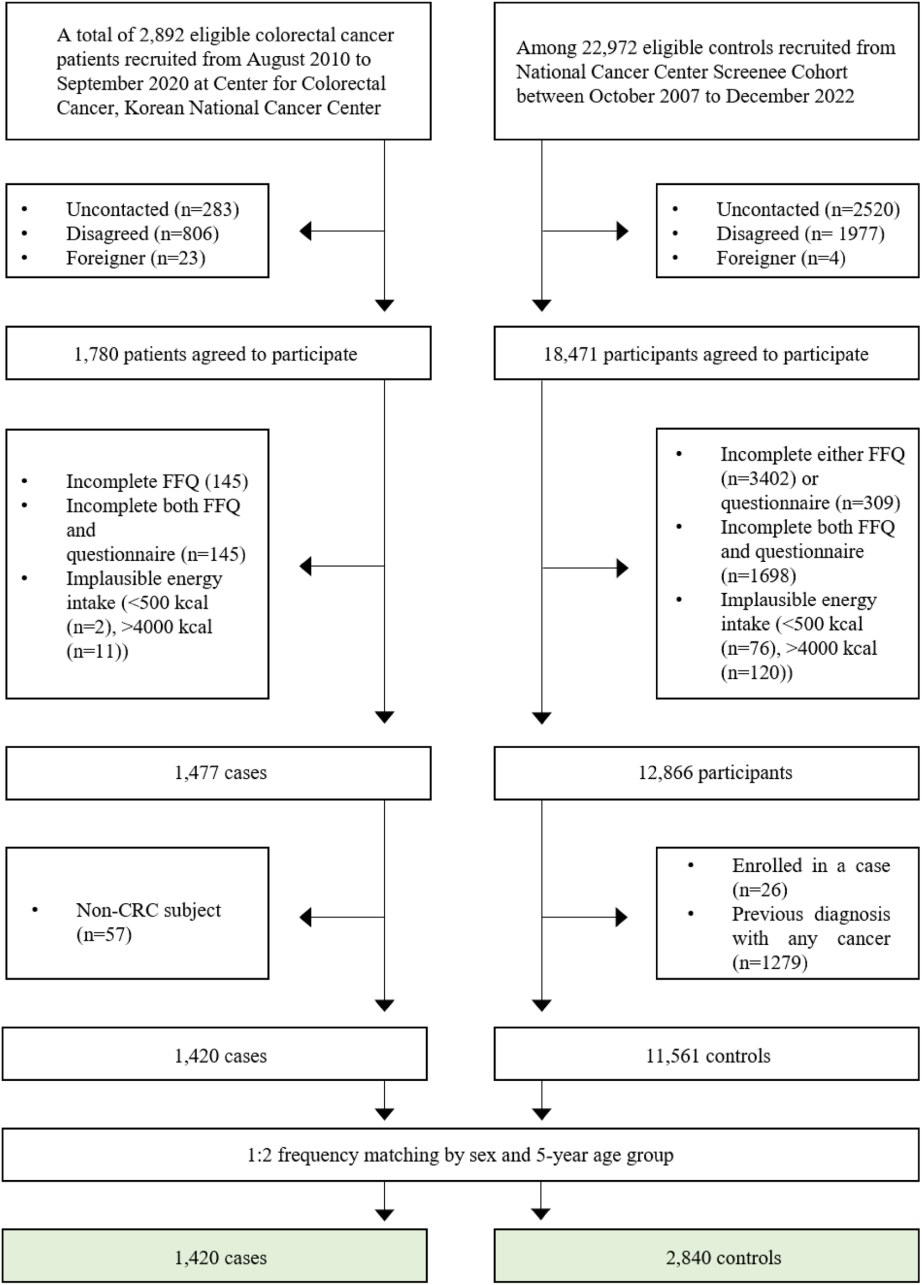
Flowchart of the study participants.

This hospital-based, case‒control study recruited participants from two research centers of the National Cancer Center (NCC) of the Republic of Korea. In the CRC patient group, people who were newly diagnosed with CRC between August 2010 and September 2020 at the Center for Colorectal Cancer of the NCC were included. Of the 1780 patients who agreed to participate in this study, 290 participants were excluded due to incomplete semiquantitative food frequency questionnaire (SQFFQ) or general questionnaire data, and 13 others were excluded due to implausible energy intake (< 500 kcal/day or > 4000 kcal/day). We also excluded 57 non-CRC patients. Thus, there were 1420 eligible CRC patients for the study. The control group consisted of people visiting the Center for Cancer Prevention and Detection at the same hospital for the health check-up program provided by the National Health Insurance Cooperation from October 2007 to December 2022. Of the 18,471 control participants, 5409 participants with incomplete SQFFQ or general questionnaire data and 196 others with implausible energy intake (< 500 kcal/day or > 4000 kcal/day) were excluded. Participants were also excluded if they were newly (*n* = 26) or previously (*n* = 1279) diagnosed with any cancer. Among the eligible participants, control participants were selected by frequency matching to CRC patients by sex and 5-years age group (case:control ratio of 1:2). Therefore, 1420 CRC patients and 2840 control participants were included in the final analysis (Fig. 1).

### Ethical approval

This study was conducted according to the guidelines laid down in the Declaration of Helsinki, and all procedures involving human subjects/patients were approved by the Institutional Review Board of Korea National Cancer Center (IRB No. NCC2021-0181). All study participants provided written informed consent before participating in the study.

### Outcome assessment

We classified CRC patients into three groups according to anatomical subsite based on the International Statistical Classification of Disease and Related Health Problems, 10th revision (ICD-10)^25^: (1) the proximal colon (including cecum, ascending colon, hepatic flexure, transverse colon, and splenic flexure); (2) the distal colon (including the descending colon, sigmoid-descending colon junction, and sigmoid colon); and (3) the rectum (including the rectosigmoid colon and rectum).

### Data collection

All participants were interviewed about their sociodemographic and lifestyle characteristics, including age, sex, weight, height, first-degree family history of CRC, marital status, education level, monthly income, occupation, smoking status, alcohol consumption, and physical activity, using a structured questionnaire. Body mass index (BMI) was calculated as body weight (kg) divided by the square of height (m^2^). Dietary data were collected using a 106-item SQFFQ developed for Korean adults. The validity and reproducibility of the SQFFQ have been described elsewhere^26^. Participants were asked to provide their average food frequency (on a 9-point scale of never or rarely, 1 time per month, 2–3 times per month, 1–2 times per week, 3–4 times per week, 5–6 times per week, 1 time per day, 2 times per day, or 3 times per day) and the average portion size (on a 3-point scale of small, medium, or large) for each food item during the previous year. The food items listed in the SQFFQ were categorized into 33 food groups based on nutritional similarities and culinary usage (Supplementary Table 1).

### Dietary pattern analysis

Dietary patterns were assessed by the PCA (PROC FACTOR) and RRR (PROC PLS) methods using 33 predefined food groups. The intake of these food groups was adjusted for total energy intake by density methods (g/1000 kcal). For the PCA method, orthogonal varimax rotation was applied to enhance the interpretability of the extracted components. We decided to retain two factors based on the eigenvalue (greater than 2.0), the inflection point of the scree plot, and the interpretability of the components. For the RRR method, we used four response variables (ratio of n−6:n−3, fiber, vitamin D, and calcium) associated with CRC^27,28^. We retained only the first factor that explained most of the variation in the response variables. For each dietary pattern, we calculated a score by summing the intakes of each food group weighted by the factor loadings ( ≥|0.20|). However, six food groups (light-colored vegetables, fish, mushrooms, other seafoods, eggs, and red meat) had factor loadings ≥|0.20| in both PCA-derived dietary patterns. For calculating dietary pattern scores, these food groups belonged to one pattern with high loadings. Moreover, we obtained sex-specific pattern scores by conducting dietary pattern analysis for men and women. Dietary patterns were named according to the food groups showing high loadings.

### Statistical analysis

The descriptive statistics are presented as the mean ± standard deviation for continuous variables and as numbers (percentages) for categorical variables. The generalized linear model and the chi-square test were used to compare the differences in the means and distributions of the general characteristics of the study participants, respectively. Dietary pattern scores were divided into quartiles based on their distribution among the control participants. The association between dietary pattern scores and CRC risk was assessed using unconditional logistic regression models to calculate odds ratios (ORs) and 95% confidence intervals (CIs). The lowest intake group (Q1) was used as the reference. The median value for each quartile category of the dietary pattern score was used as a continuous variable to test for trends in the regression model. The multivariable logistic regression model considered potential covariates such as age (continuous), BMI (continuous), first-degree family history of CRC (yes/no), marital status (married/others), education level (elementary school or less, middle school, high school, and college or more), monthly income (< 2, 2–4, ≥ 4 million won per month), occupation (professionals/administrative management/office jobs, sales/service, agriculture/manufacturing/mining/army service, and housekeeping/unemployment/others), smoking status (nonsmoker, former smoker, and current smoker), alcohol consumption (nondrinker, former drinker, and current drinker), and regular physical activity (yes/no). Stratified analysis based on anatomical subsites (proximal colon, distal colon, and rectal cancers) was conducted using multinomial logistic regression models. All analyses were performed using SAS version 9.4 (SAS Institute, Inc., Cary, NC, USA). Statistical significance was considered at *P* < 0.05.

## Results

### General characteristics of the study population

The general characteristics of the study population are described in Table 1. The mean age was 58.1 ± 10.2 years in the CRC patient group and 57.6 ± 9.4 years in the control group. Among the overall population, CRC patients had higher rates of first-degree family history of CRC and married status, a greater proportion of former drinkers, and higher energy intake than control participants (*P* < 0.05). Moreover, participants in the CRC patient group had lower levels of education, monthly income, professional occupation status, and physical activity than those in the control group (*P* < 0.001). When stratified by sex, compared with control participants, male CRC patients had a significantly lower mean BMI and smoking rate, and female CRC patients had a significantly higher mean BMI (*P* < 0.05). The same trend was observed in the distribution of other characteristics for both men and women (*P* < 0.05).

**Table 1:** General characteristics of the study population.

Variables	Total			Men			Women		
	Control (n = 2840)	Case (n = 1420)	*P*	Control (n = 1832)	Case (n = 916)	*P*	Control (n = 1008)	Case (n = 504)	*P*
Age (years)	57.6 ± 9.4	58.1 ± 10.2	0.099	57.8 ± 9.0	58.5 ± 9.9	0.107	57.0 ± 10.1	57.4 ± 10.7	0.524
Body mass index (kg/m^2^)									
Mean	24.2 ± 2.9	24.0 ± 3.4	0.103	24.5 ± 2.7	24.0 ± 3.1	< 0.001	23.6 ± 3.0	24.0 ± 3.8	0.028
< 18.5	48(1.7)	44(3.1)	< 0.001	24(1.3)	24(2.6)	< 0.001	24(2.4)	20(3.9)	0.009
18.5–< 23	926(33.0)	528(37.2)		495(27.4)	323(35.3)		431(43.1)	205(40.7)	
23–< 25	778(27.8)	335(23.6)		522(28.9)	233(25.5)		256(25.6)	102(20.2)	
≥ 25	1047(37.4)	509(35.9)		760(42.2)	333(36.4)		287(28.7)	176(34.9)	
First-degree family history of colorectal cancer	150 (5.2)	145 (10.2)	< 0.001	86 (4.6)	98 (10.7)	< 0.001	64 (6.3)	47 (9.3)	0.036
Marital status									
Married	2404 (85.5)	1243 (87.8)	0.038	1637 (89.9)	830 (90.8)	0.497	767 (77.3)	413 (82.4)	0.021
Others: single, divorced, separated, widowed, cohabitating	407 (14.4)	172 (12.1)		182 (10.1)	84 (9.1)		225 (22.6)	88 (17.5)	
Education									
≤ Elementary school	174(6.2)	253(17.8)	< 0.001	77(4.2)	114(12.4)	< 0.001	97(9.7)	139(27.6)	< 0.001
Middle school	205(7.3)	204(14.3)		127(7.0)	135(14.7)		78(7.8)	69(13.7)	
High school	1184(42.3)	592(41.7)		710(39.4)	395(43.1)		474(47.4)	197(39.2)	
≥ College	1236(44.1)	369(26.0)		886(49.2)	272(29.6)		350(35.0)	97(19.3)	
Monthly income (10,000 won/month)									
< 200	652(23.6)	561(39.8)	< 0.001	365(20.5)	359(39.4)	< 0.001	287(29.4)	202(40.5)	< 0.001
200–400	1075(39.0)	518(36.8)		718(40.4)	330(36.3)		357(36.5)	188(37.7)	
≥ 400	1025(37.2)	328(23.3)		693(39.0)	220(24.2)		332(34.0)	108(21.6)	
Occupation									
Group 1: professionals, administrative management, office jobs	792 (28.1)	332 (23.4)	< 0.001	604 (33.3)	268 (29.2)	< 0.001	188 (18.8)	64 (12.7)	< 0.001
Group 2: sales and service positions	574 (20.4)	90 (6.3)		437 (24.1)	60 (6.5)		137 (13.7)	30 (5.9)	
Group 3: agriculture, manufacturing, mining, army service	376 (13.3)	168 (11.8)		328 (18.1)	144 (15.7)		48 (4.8)	24 (4.7)	
Group 4: housekeeping, unemployment, and others	1068 (38.0)	829 (58.4)		441 (24.3)	444 (48.4)		627 (62.7)	385 (76.5)	
Smoking status									
Never	1311(46.1)	689(48.5)	0.318	372(20.3)	234(25.5)	0.007	939(93.1)	455(90.4)	0.149
Past	1058(37.2)	500(35.2)		1014(55.3)	472(51.5)		44(4.3)	28(5.5)	
Current	471(16.5)	230(16.2)		446(24.3)	210(22.9)		25(2.4)	20(3.9)	
Alcohol consumption									
Never	858(30.2)	530(37.3)	< 0.001	306(16.7)	204(22.2)	< 0.001	552(54.7)	326(64.8)	< 0.001
Past	272(9.5)	204(14.3)		227(12.3)	161(17.5)		45(4.4)	43(8.5)	
Current	1710(60.2)	685(48.2)		1299(70.9)	551(60.1)		411(40.7)	134(26.6)	
Regular physical activity									
Yes	1589 (59.7)	504 (35.4)	< 0.001	1070 (59.3)	344 (37.5)	< 0.001	519 (58.4)	160 (31.7)	< 0.001
No	1101 (40.9)	916 (64.5)		732 (40.6)	572 (62.4)		369 (41.5)	344 (68.2)	
Total energy intake (kcal/day)	1741.1 ± 567.0	2043.5 ± 575.2	< 0.001	1785.1 ± 547.7	2162.5 ± 542.5	< 0.001	1661.1 ± 592.3	1827.2 ± 570.4	< 0.001

The values are expressed as the means (SDs) or numbers (percentages).

### Dietary patterns

The factor loadings of dietary patterns determined by the PCA and RRR methods are shown in Table 2. PCA identified two major dietary patterns among men and women: a prudent pattern and a westernized pattern. The first dietary pattern (“prudent” pattern) derived from PCA was mainly characterized by a high consumption of green/yellow vegetables, condiments/seasonings, light-colored vegetables, tubers, seaweeds, fish, mushrooms, fruits, tofu/soymilk, other seafoods, kimchi, eggs, dairy products, nuts, pickled vegetables, and legumes for both men and women. Milk was also represented in the prudent dietary pattern among women. The second dietary pattern (“Westernized” pattern) derived from PCA was characterized by a high consumption of red meat, oil, sweets, noodles, processed meat, meat byproducts, poultry, carbonated beverages, bread/cake/pizza/hamburgers, seafood products, salted and fermented seafood, cereals and snacks for both men and women. The factor loadings from RRR seemed to be similar to those of the prudent dietary pattern. As in the prudent dietary pattern, the RRR-derived dietary pattern (“healthy” pattern) was characterized by higher consumption of green/yellow vegetables, light-colored vegetables, fruits, eggs, and milk for both men and women. In addition, kimchi was represented in the healthy dietary pattern among men.

**Table 2:** Factor loading matrix for the 3 major patterns identified by factor analysis.

	Men			Women		
	PCA		RRR	PCA		RRR
	Pattern 1	Pattern 2		Pattern 1	Pattern 2	
Food groups						
Green/yellow vegetables	0.77	− 0.01	0.23	0.81	− 0.06	0.23
Condiments/seasonings	0.79	0.00	− 0.04	0.80	− 0.01	− 0.04
Light-colored vegetables	0.70	0.29	0.20	0.77	0.19	0.22
Tubers	0.61	0.01	0.11	0.64	0.03	0.10
Seaweeds	0.59	0.07	0.08	0.59	0.02	0.04
Fish	0.56	0.17	0.12	0.54	0.20	0.15
Mushrooms	0.48	0.27	0.06	0.49	0.17	0.04
Fruits	0.41	0.01	0.26	0.52	− 0.06	0.31
Tofu/soymilk	0.46	0.03	0.12	0.55	0.01	0.12
Other seafoods	0.48	0.34	0.07	0.50	0.30	0.06
Kimchi	0.39	0.10	0.25	0.35	0.07	0.19
Eggs	0.24	0.21	0.48	0.40	0.14	0.48
Dairy products	0.27	0.07	0.11	0.32	0.05	0.11
Nuts	0.27	− 0.05	0.03	0.32	− 0.13	0.01
Pickled vegetables	0.31	0.03	0.04	0.25	0.10	0.02
Milk	0.18	0.01	0.31	0.30	− 0.05	0.27
Legumes	0.27	− 0.10	0.16	0.28	− 0.01	0.17
Red meat	0.22	0.62	0.03	0.34	0.54	− 0.00
Coffee/tea	0.17	0.08	0.07	0.30	0.09	0.05
Oil	0.04	0.55	0.00	0.10	0.59	− 0.01
Sweets	0.03	0.48	0.02	0.06	0.51	0.03
Noodles	0.03	0.51	0.07	0.13	0.50	0.05
Processed meat	− 0.01	0.54	0.00	− 0.07	0.46	− 0.00
Meat byproducts	0.13	0.46	− 0.00	0.14	0.44	0.02
Poultry	0.14	0.40	0.00	0.02	0.49	0.00
Carbonated beverages	− 0.03	0.40	0.00	− 0.04	0.26	0.00
Bread/cake/pizza/hamburgers	0.02	0.37	0.12	0.09	0.50	0.12
Seafood products	0.16	0.37	− 0.00	0.14	0.39	− 0.03
Salted and fermented seafoods	0.13	0.26	0.02	0.08	0.32	0.00
Cereals and snacks	− 0.01	0.36	0.01	0.01	0.39	0.02
Refined grains	0.06	0.07	− 0.01	− 0.03	0.11	− 0.01
Rice cakes	0.15	0.12	0.02	0.08	0.15	0.01
Whole grains	0.12	− 0.21	0.06	0.04	− 0.07	0.05
Proportion of variance explained by food groups or predictors (%)	15.44	7.08	12.67	16.80	7.51	14.45
Proportion of variance explained by responses (%)			46.11			49.93
Ratio of n−6:n−3	–	–	0.15	–	–	0.57
Fiber	–	–	68.37	–	–	74.73
Vitamin D	–	–	41.22	–	–	48.83
Calcium	–	–	74.71	–	–	75.58

As expected, the percentage of variation explained by food groups or predictors was higher for the PCA-derived pattern in both men and women (men: 15.44% in PCA 1 vs. 12.67% in RRR-derived pattern; women: 16.80% in PCA 1 vs. 14.45% in RRR-derived pattern). In the RRR pattern, the explained variation in responses was 46.11% for men and 49.93% for women.

### Associations between dietary patterns and CRC risk

The associations between dietary pattern scores and CRC risk are presented in Tables 3 and 4. In men, a significant inverse association between dietary pattern and risk of rectal cancer was found only for the healthy dietary pattern (OR_Q4 vs. Q1_ = 0.66, 95% CI 0.45–0.97, *P* for trend = 0.036) after adjustment for age, BMI, first-degree family history of CRC, marital status, education, monthly income, occupation, smoking status, alcohol consumption, and regular physical activity. In women, the risk of CRC tended to decrease for the highest quartile of prudent and healthy dietary patterns (prudent, OR_Q4 vs. Q1_ = 0.59, 95% CI 0.40–0.86, *P* for trend = 0.005; healthy, OR_Q4 vs. Q1_ = 0.62, 95% CI 0.43–0.89, *P* for trend = 0.007) after adjustment for confounding factors. According to an analysis stratified by anatomical subsite, a decreased risk of rectal cancer was observed for those in the highest quartile of the prudent dietary pattern (OR_Q4 vs. Q1_ = 0.31, 95% CI 0.15–0.65, *P* for trend < 0.001). A healthy dietary pattern was associated with a decreased risk of distal colon cancer (OR_Q4 vs. Q1_ = 0.58, 95% CI 0.35–0.97, *P* for trend = 0.025) and rectal cancer (OR_Q4 vs. Q1_ = 0.29, 95% CI 0.15–0.57, *P* for trend < 0.001). The Westernized dietary pattern was not significantly associated with the risk of CRC in either sex.

**Table 3:** Colorectal cancer risk according to anatomical location and dietary intake of the identified dietary patterns (men).

		Colorectal cancer			Proximal colon cancer			Distal colon cancer					
	No.controls	No.cases	Unadjusted OR(95% CI)	Multivariable OR(95% CI) ^†^	No.cases	Unadjusted OR(95% CI)	Multivariable OR(95% CI) ^†^	No.cases	Unadjusted OR(95% CI)	Multivariable OR(95% CI) ^†^	No.cases	Unadjusted OR(95% CI)	Multivariable OR(95% CI)^†^
PCA- 1: (prudent)													
Q1	458	198	1.00 (reference)	1.00 (reference)	58	1.00 (reference)	1.00 (reference)	61	1.00 (reference)	1.00 (reference)	76	1.00 (reference)	1.00 (reference)
Q2	457	312	1.57 (1.26–1.96)	1.65 (1.28–2.13)	74	1.27 (0.88–1.84)	1.33 (0.90–1.99)	91	1.49 (1.05–2.12)	1.59 (1.09–2.31)	138	1.82 (1.33–2.47)	1.91 (1.36–2.69)
Q3	458	233	1.17 (0.93–1.48)	1.28 (0.98–1.67)	71	1.22 (0.84–1.77)	1.31 (0.88–1.96)	70	1.14 (0.79–1.65)	1.22 (0.82–1.81)	88	1.15 (0.83–1.61)	1.29 (0.89–1.86)
Q4	459	173	0.87 (0.68–1.10)	1.00 (0.75–1.34)	65	1.11 (0.76–1.63)	1.23 (0.81–1.87)	56	0.91 (0.62–1.34)	1.00 (0.65–1.52)	51	0.67 (0.45–0.97)	0.84 (0.55–1.29)
p for trend			0.013	0.317		0.773	0.445		0.226	0.475		< 0.001	0.083
PCA- 2: (Westernized)													
Q1	457	191	1.00 (reference)	1.00 (reference)	56	1.00 (reference)	1.00 (reference)	57	1.00 (reference)	1.00 (reference)	73	1.00 (reference)	1.00 (reference)
Q2	458	180	1.46 (1.16–1.83)	1.66 (1.28–2.17)	70	1.24 (0.85–1.81)	1.47 (0.98–2.21)	87	1.52 (1.06–2.17)	1.73 (1.18–2.55)	119	1.62 (1.18–2.23)	1.75 (1.23–2.51)
Q3	459	231	1.20 (0.95–1.51)	1.35 (1.02–1.78)	71	1.26 (0.86–1.83)	1.45 (0.96–2.19)	71	1.24 (0.85–1.79)	1.41 (0.94–2.12)	84	1.14 (0.81–1.60)	1.20 (0.82–1.76)
Q4	458	214	1.11 (0.88–1.41)	1.22 (0.92–1.63)	71	1.26 (0.87–1.83)	1.49 (0.98–2.28)	63	1.10 (0.75–1.61)	1.22 (0.80–1.87)	77	1.05 (0.74–1.48)	1.05 (0.70–1.57)
p for trend			0.952	0.722		0.286		0.123	0.854	0.845		0.460	0.419
RRR (healthy)													
Q1	458	248	1.00 (reference)	1.00 (reference)	52	1.00 (reference)	1.00 (reference)	77	1.00 (reference)	1.00 (reference)	112	1.00 (reference)	1.00 (reference)
Q2	458	264	1.06 (0.85–1.32)	1.06 (0.82–1.36)	77	1.48 (1.01–2.15)	1.50 (1.00–2.25)	75	0.97 (0.69–1.37)	1.02 (0.71–1.48)	104	0.92 (0.69–1.24)	0.91 (0.66–1.27)
Q3	457	214	0.86 (0.69–1.08)	0.96 (0.74–1.25)	73	1.40 (0.96–2.05)	1.51 (1.00–2.28)	59	0.76 (0.53–1.10)	0.84 (0.57–1.24)	82	0.73 (0.53–1.00)	0.87 (0.61–1.23)
Q4	459	190	0.76 (0.60–0.96)	0.96 (0.73–1.25)	66	1.26 (0.86–1.86)	1.57 (1.03–2.39)	67	0.86 (0.61–1.23)	1.02 (0.69–1.50)	55	0.49 (0.34–0.69)	0.66 (0.45–0.97)
p for trend			0.005	0.630		0.505	0.072		0.330	0.966		< 0.001	0.036

^†^Adjusted for body mass index, first-degree family history of colorectal cancer, marital status, education, monthly income, occupation, smoking status, alcohol consumption, regular physical activity, and matching factor.

**Table 4:** Colorectal cancer risk according to anatomical location and dietary intake of the identified dietary patterns (women).  Significant values are in bold.

	No.controls	Colorectal cancer			Proximal colon cancer			Distal colon cancer			Rectal cancer		
		No.cases	Unadjusted OR(95% CI)	Multivariable OR(95% CI) ^†^	No.cases	Unadjusted OR(95% CI)	Multivariable OR(95% CI) ^†^	No.cases	Unadjusted OR(95% CI)	Multivariable OR(95% CI) ^†^	No.casess	Unadjusted OR(95% CI)	Multivariable OR(95% CI) ^†^
PCA- 1: (prudent)													
Q1	251	169	1.00 (reference)	1.00 (reference)	47	1.00 (reference)	1.00 (reference)	63	1.00 (reference)	1.00 (reference)	55	1.00 (reference)	1.00 (reference)
Q2	252	148	0.87 (0.65–1.15)	0.91 (0.65–1.27)	44	0.93 (0.59–1.45)	0.90 (0.55–1.47)	46	0.72 (0.47–1.10)	0.78 (0.49–1.24)	56	1.01 (0.67–1.53)	1.10 (0.70–1.74)
Q3	252	115	0.67 (0.50–0.91)	0.80 (0.56–1.13)	48	1.01 (0.65–1.57)	1.07 (0.66–1.74)	35	0.55 (0.35–0.86)	0.65 (0.39–1.06)	30	0.54 (0.33–0.87)	0.69 (0.41–1.17)
Q4	253	72	0.42 (0.30–0.58)	0.59 (0.40–0.86)	29	0.61 (0.37–1.00)	0.70 (0.40–1.21)	32	0.50 (0.31–0.79)	0.75 (0.44–1.26)	11	0.19 (0.10–0.38)	0.31 (0.15–0.65)
p for trend		< 0.001	**0.005**		0.072	0.305		0.001	0.183		< 0.001	** < 0.001**	
PCA- 2: (Westernized)													
Q1	252	135	1.00 (reference)	1.00 (reference)	57	1.00 (reference)	1.00 (reference)	45	1.00 (reference)	1.00 (reference)	30	1.00 (reference)	1.00 (reference)
Q2	251	141	1.04 (0.78–1.40)	1.26 (0.89–1.79)	40	0.70 (0.45–1.09)	0.85 (0.52–1.38)	48	1.07 (0.68–1.66)	1.29 (0.79–2.11)	52	1.74 (1.07–2.81)	2.05 (1.20–3.50)
Q3	253	116	0.85 (0.63–1.15)	0.96 (0.67–1.37)	41	0.71 (0.46–1.11)	0.95 (0.58–1.54)	38	0.84 (0.52–1.34)	0.86 (0.51–1.45)	37	1.22 (0.73–2.05)	1.23 (0.70–2.18)
Q4	252	112	0.83 (0.61–1.12)	0.94 (0.65–1.37)	30	0.52 (0.32–0.84)	0.76 (0.44–1.30)	45	1.00 (0.63–1.56)	0.99 (0.58–1.67)	33	1.10 (0.65–1.85)	1.08 (0.59–1.97)
p for trend		0.127	0.453		0.010	0.356		0.826	0.674		0.722	0.580	
RRR (healthy)													
Q1	251	186	1.00 (reference)	1.00 (reference)	46	1.00 (reference)	1.00 (reference)	71	1.00 (reference)	1.00 (reference)	65	1.00 (reference)	1.00 (reference)
Q2	253	130	0.69 (0.52–0.92)	0.72 (0.51–1.01)	42	0.90 (0.57–1.42)	0.89 (0.54–1.47)	43	0.60 (0.39–0.91)	0.61 (0.38–0.97)	43	0.65 (0.43–1.00)	0.73 (0.45–1.17)
Q3	251	97	0.52 (0.38–0.70)	0.58 (0.41–0.83)	34	0.73 (0.45–1.19)	0.76 (0.45–1.28)	30	0.42 (0.26–0.67)	0.48 (0.29–0.79)	31	0.47 (0.30–0.75)	0.55 (0.33–0.92)
Q4	253	91	0.48 (0.35–0.65)	0.62 (0.43–0.89)	46	0.99 (0.63–1.54)	1.07 (0.65–1.77)	32	0.44 (0.28–0.70)	0.58 (0.35–0.97)	13	0.19 (0.10–0.36)	0.29 (0.15–0.57)
p for trend		< 0.001	0.007		0.953	0.766		< 0.001	0.025		< 0.001	< 0.001	

## Discussion

In the present study, we used both PCA and RRR to investigate the association between dietary patterns and CRC risk in Korean adults. Using PCA, two dietary patterns were derived from men and women: a prudent pattern and a westernized pattern. A prudent dietary pattern was associated with a decreased risk of CRC after adjustment for confounding factors among women only. The westernized dietary pattern was not significantly associated with CRC in either sex. Using RRR analysis, we identified a dietary pattern that seemed to be similar to the prudent dietary pattern in both men and women. A significant inverse association was observed between a healthy pattern and rectal cancer risk in men. In women, a healthy dietary pattern was associated with a significantly lower risk of CRC, as well as distal colon and rectal cancer.

Our findings showed that higher adherence to the prudent dietary pattern decreased CRC risk in women. The prudent dietary pattern was characterized by high intakes of green/yellow vegetables, condiments/seasonings, light-colored vegetables, tubers, seaweeds, fish, mushrooms, fruits, tofu/soymilk, other seafoods, kimchi, eggs, dairy products, nuts, pickled vegetables, milk, and legumes. One prospective cohort analysis from Alberta’s Tomorrow Project indicated that a PCA-derived, prudent dietary pattern was protective against combined cancer and colon cancer^22^. A healthy dietary pattern that was high in vegetables, fruits and fish was reported to be inversely associated with CRC risk in a European cohort study among women^29^. In contrast, the results from the Dietary Patterns and Cancer Project reported that there is no association between a vegetable-based pattern and CRC risk^30^. Consistent with our results, a case‒control study in Korea showed that a prudent dietary pattern rich in fruit and dairy products was inversely associated with CRC risk^12^. Another case‒control study conducted in central and northeast Pennsylvania reported that higher scores on the fruits and vegetables pattern were associated with a reduced risk of CRC^18^.

The beneficial effects of the prudent dietary pattern on CRC prevention might be explained by numerous mechanisms. The prudent diet is rich in various plant-based foods, such as fruits and vegetables, which contain antioxidant vitamins, carotenoids, fiber, folic acid, and other phytochemical compounds^31^. Antioxidant micronutrients, including vitamin C, vitamin E, and carotenoids, trap free radicals and reactive oxygen species^32^. Specifically, carotenoids are efficient scavengers of reactive oxygen species and stimulate the immune system^33^. Vitamin C reduces nitrite, thus blocking the formation of nitrosamines and nitrosamides, which are known carcinogens that contribute to the induction of tumors in experimental animals and possibly in humans^34^. Several nitrosamides have been shown to induce CRC and adenomatous polyps in the large intestine of rats when applied directly to the colonic epithelium^35^. Dietary fiber may dilute carcinogens through increased stool bulk and decrease the contact time of carcinogens and toxins with the colonic epithelium due to reduced transit time^36^. Moreover, the decreased fecal pH caused by dietary fiber inhibits bacterial degradation of normal food constituents to potential carcinogens^37^. Fiber fermentation by fecal flora to short-chain fatty acids (SCFAs), such as acetate, propionate, and butyrate, is known to be the key factor in the suppression of colonic inflammation and carcinogenesis^38^. SCFAs have anticancer effects, including the promotion of apoptosis in cancer cells^39^ and the inhibition of chronic inflammatory processes and cancer cell migration/invasion in the colon^40^. A number of such metabolites related to dietary intake could be an important way to reflect the components of food as well as markers for complex metabolomics responses to dietary exposures. One study that developed metabolite profile scores correlated with dietary intake and also examined the prospective associations of these scores with CRC risk, showed consistent results compared to previous studies using dietary data only^41^. Our future research could benefit from the incorporation of metabolomics to complement traditional dietary assessments in investigating the diet‒CRC association.

Several cohort studies have shown a protective effect of dairy product and milk intake on the risk of CRC^42^. A case‒control study conducted in China revealed that subjects in the highest quartile had a 68% and 48% lower risk of CRC than those in the lowest quartile of total dairy product and milk consumption, respectively^43^. There are several mechanisms by which dairy products and milk are related to a reduction in CRC risk. Dairy products include beneficial constituents, such as butyric acid, lactoferrin, and fermentation products^44^. Milk is a rich source of dietary calcium and vitamin D. Vitamin D may protect against CRC since it reduces epithelial cell proliferation and exerts anticancer effects^45,46^. Furthermore, the roles of calcium and vitamin D are closely linked because vitamin D is involved in the regulation of calcium bioavailability^47^.

In the present study, no associations were found between the Westernized dietary pattern and CRC risk in either men or women. In Western countries, most studies have shown a positive association between the Western dietary pattern and CRC^48–50^. On the other hand, a cohort study conducted in Japan reported that an animal food dietary pattern characterized by a high intake of various animal-derived foods, such as beef, pork, ham, sausage, poultry, liver, and butter, was not significantly associated with a risk of CRC^51^. In another cohort of Singaporean Chinese individuals, the meat-dim sum pattern which was similar to the Western dietary pattern, was not associated with CRC^52^. The differences among studies may be partially attributed to different amounts and ranges of meat intake and various cooking methods, such as grilling or stir frying between Western and Asian populations^53,54^.

Our study showed that a healthy dietary pattern was inversely associated with CRC risk in women. A healthy dietary pattern was characterized by high consumption of green/yellow vegetables, light-colored vegetables, fruits, eggs, and milk. This pattern is somewhat similar to the prudent pattern. A previous study also used RRR analysis with nutrients as a response variable, and the dietary fiber pattern and discretionary fats pattern were shown to be protective against colon cancer^22^. Another study using RRR analysis showed that secondary fecal bile acid concentrations increased across the tertiles of dietary pattern, which was characterized by high intakes of processed meat, fried potatoes, bread, and margarine and low intakes of muesli, plant-based milk, vegetables, and fruit^55^. Previous research has indicated that an increased proportion of secondary bile acids in feces is present in patients with CRC^56^.

Sex differences were also observed in our study. Generally, women have been reported to engage in more health-promoting behaviors than men and have healthier lifestyle patterns. According to our prudent and healthy patterns, the loading of plant-based foods, such as fruits and vegetables, was higher among women than among men^57^. Moreover, diet and dietary patterns play critical roles in obesity, which causes low-grade chronic inflammation^58^. Chronic inflammation is one of the major predisposing factors in cancer progression and is indicated by increased plasma levels of C-reactive protein (CRP), interleukin 6, and tumor necrosis factor-alpha. A meta-analysis revealed that prediagnostic circulating CRP concentrations were positively associated with the risk of CRC, and the association was stronger in men than in women^59^. These sex-dependent variations might affect the reduced risk of CRC in females compared to males.

Considering that dietary patterns generated by PCA reflect real-world dietary habits in a population and that dietary patterns generated by RRR are associated with the disease of interest, patterns derived from each method could be different. However, we identified two similar dietary patterns using PCA and RRR analysis. Moreover, compared to the dietary pattern determined by PCA, the RRR-derived pattern had a slightly stronger association with CRC in women. Similarly, several studies have shown that RRR patterns are more strongly associated with outcomes than PCA patterns because dietary patterns from the RRR method are driven via disease-associated response variables^60–62^. In contrast, a case‒control study investigating the associations between dietary patterns and bladder cancer showed stronger associations for PCA patterns^63^. Meanwhile, each of these methods has drawbacks. PCA is unable to account for which dietary patterns have the most predictive capability for a disease^64^. In terms of choosing the most intermediate response variables for the RRR analysis based on prior knowledge, relying solely on the information of selected response variables to derive dietary patterns may lead to the omission of those dietary patterns related to nutrients in the disease’s metabolic pathways but not included in the response variables. Therefore, the PCA and RRR methods complement each other, and the application of both methods might be useful for assessing the similarity between actual dietary behavior and disease-associated patterns.

The main strength of the current study was the application of both PCA and RRR analysis to compare dietary patterns derived from those two methods in a relatively large population. Second, we conducted a risk-stratified analysis of the association between dietary patterns and distinct CRC locations for each sex. Third, we had comprehensive information on potential confounding variables based on a questionnaire administered by skilled interviewers. However, there are several limitations that need to be acknowledged when interpreting the results. Selection bias is an inherent potential limitation of case‒control studies. Control participants were recruited from those who enrolled in a health screening program. Thus, participants in the control group might have had healthier behaviors compared with individuals in the general population. In addition, there could be recall bias in reporting dietary consumption since dietary intake was assessed using the SQFFQ. However, the previously validated SQFFQ used in this study was designed to collect data regarding the usual dietary intake of the Korean population. Additionally, trained interviewers assisted participants with the structured questionnaire to minimize this recall bias. Finally, some subjective decisions were made regarding dietary pattern analyses, such as food grouping, labeling of patterns, and choosing intermediate response variables for the RRR, which might lead to the omission of relevant nutrients in the biological pathways involved in CRC.

In conclusion, we obtained two similar patterns using PCA and RRR analysis. Although both patterns were associated with a lower risk of CRC, the healthy dietary pattern showed a slightly stronger association in women. The concordant food groups in the prudent and healthy dietary patterns consisted of green/yellow vegetables, light-colored vegetables, fruits, eggs, and milk. In this particular study, the RRR-derived dietary pattern was strongly associated with CRC risk in the study population and might be more suitable for deriving the pattern that is associated with CRC. However, further investigations are needed in different populations and with different response variables and other disease outcomes.

### Supplementary Information

Supplementary Table 1.

## Data Availability

The data used and analyzed during the current study are available from the corresponding author upon reasonable request.
