# Supplementary material for: Sex-specific associations of empirically derived dietary patterns with colorectal cancer risk in a Korean population: a case‒control study
Source: Sci Rep. 2024 Mar 20;14:6709. doi: 10.1038/s41598-024-55524-5 (PMC10954725; doi:10.1038/s41598-024-55524-5)
Supplement: Supplementary file 1 — Supplementary Table 1. [file 41598_2024_55524_MOESM1_ESM.docx]

| **Food group** | **Dietary components** |
| --- | --- |
| Whole grains | Glutinous rice, brown rice, barley, black rice, foxtail millet, sorghum |
| Refined grains | Wheat flour, white rice |
| Noodles | Ramyun (instant noodle), Chinese noodle, udong, wheat noodle |
| Bread/cake/pizza/hamburgers | Bread, pizza, hamburger, sandwich, cake, pie, doughnut |
| Cereals and snacks | Cereals, rice crispy, corn flakes, snack, biscuit, cookies, cracker |
| Rice cakes | Rice cakes |
| Tubers | Potatoes, sweet potatoes, potato starch, cellophane noodles |
| Sweets | Honey, sugar, starch syrup, candies, caramel, chocolates, fruit jams |
| Legumes | Soybeans, green peas, black beans |
| Tofu/soymilk | Tofu, soft tofu, fried tofu, soybean curd residue, soy milk |
| Nuts | Peanuts, almonds, pine nut, sesames |
| Green/yellow vegetables | Green chilli, red chilli, red-pepper leaf, leaf beet, carrots, spinach, lettuce, leeks, broccoli, tomatoes, tomato juice, tomato paste, green onion, pumpkin, zucchini, Korean lettuce, squash |
| Light-colored vegetables | Garlic, radishes, ginger, celery, brussels sprouts, onion, cucumber, bean sprouts |
| Pickled vegetables | Pickled radishes, pickled cucumber, pickled garlic |
| Kimchi | Korean cabbage, Korean cabbage (non-red pepper), seasoned cubed radish roots, na-bak, dong-chi-mi |
| Mushrooms | Oyster mushroom, matsutake mushroom, button mushroom, oak mushroom, winter fungus |
| Fruits | Strawberries, oriental melon, melon, watermelon, peaches, plum, bananas, persimmons, tangerine, pears, apples, oranges, grapes, fruit juices, canned fruits |
| Red meat | Beef (loin, tender loin, ribs), pork (loin, tender loin, shoulder. ribs, belly) |
| Meat by-products | Beef (small intestine), pork (feet) |
| Processed meat | Ham, bacon, sausages |
| Poultry | Chicken |
| Eggs | Eggs, quail's eggs |
| Fish | Fatty fish (mackerel, pacific saury, spanish macherel, tuna), lean fish (scabbard fish, bastard halibut, Alaska pollack, yellow corvina, bone fish (anchovy) |
| Salted and fermented seafoods | Salted fish, salted clams, pickled shrimps, fermented squid |
| Seafood products | Fish pastes |
| Other seafoods | Clam, oyster, cockle, mussel, conch, crab, shrimp, small octopus, squid |
| Seaweeds | Laver, sea tangle, sea mustard |
| Milk | Whole milk, low-fat milk, skim milk |
| Dairy products | Liquid yogurt, curd yogurt, cheese, ice cream, sherbet |
| Oil | Butter, margarine, sesame oil, soybean oil, coffee cream |
| Carbonated beverages | Soda |
| Coffee/tea | Instant coffee, green tea, citron tea, ginseng tea, sweet rice drink |
| Condiments/seasonings | Soy sauce, mustard, red pepper powder, red pepper paste, sesame salt, soybean paste, salt, vinegar, tomato ketchup, pepper powder |

**Supplementary Table 1.** Food grouping and their components.
